# Risk prediction of death and heart transplantation in adult patients with myocarditis

**DOI:** 10.1093/ehjopen/oeag104

**Published:** 2026-06-10

**Authors:** Anna Baritussio, Andrea Silvio Giordani, Chiara Merola, Giulia Lorenzoni, Cristina Vicenzetto, Federico Scognamiglio, Cristina Basso, Stefania Rizzo, Monica De Gaspari, Elisa Carturan, Giuseppe Tarantini, Sabino Iliceto, Renzo Marcolongo, Dario Gregori, Alida Linda Patrizia Caforio

**Affiliations:** Cardiology, Department of Cardiac, Thoracic, Vascular Sciences and Public Health, Padua University Hospital and University of Padua, Via Giustiniani 2, Padua 35128, Italy; Cardiology, Department of Cardiac, Thoracic, Vascular Sciences and Public Health, Padua University Hospital and University of Padua, Via Giustiniani 2, Padua 35128, Italy; Cardiology, Department of Cardiac, Thoracic, Vascular Sciences and Public Health, Padua University Hospital and University of Padua, Via Giustiniani 2, Padua 35128, Italy; Unit of Biostatistics, Epidemiology and Public Health, Department of Cardiac, Thoracic, Vascular Sciences and Public Health, Padua University Hospital and University of Padua, Via Giustiniani 2, Padua 35128, Italy; Cardiology, Department of Cardiac, Thoracic, Vascular Sciences and Public Health, Padua University Hospital and University of Padua, Via Giustiniani 2, Padua 35128, Italy; Cardiology, Department of Cardiac, Thoracic, Vascular Sciences and Public Health, Padua University Hospital and University of Padua, Via Giustiniani 2, Padua 35128, Italy; Cardiovascular Pathology, Department of Cardiac, Thoracic, Vascular Sciences and Public Health, Padua University Hospital and University of Padua, Via Giustiniani 2, Padua 35128, Italy; Cardiovascular Pathology, Department of Cardiac, Thoracic, Vascular Sciences and Public Health, Padua University Hospital and University of Padua, Via Giustiniani 2, Padua 35128, Italy; Cardiovascular Pathology, Department of Cardiac, Thoracic, Vascular Sciences and Public Health, Padua University Hospital and University of Padua, Via Giustiniani 2, Padua 35128, Italy; Cardiovascular Pathology, Department of Cardiac, Thoracic, Vascular Sciences and Public Health, Padua University Hospital and University of Padua, Via Giustiniani 2, Padua 35128, Italy; Cardiology, Department of Cardiac, Thoracic, Vascular Sciences and Public Health, Padua University Hospital and University of Padua, Via Giustiniani 2, Padua 35128, Italy; Cardiology, Department of Cardiac, Thoracic, Vascular Sciences and Public Health, Padua University Hospital and University of Padua, Via Giustiniani 2, Padua 35128, Italy; Cardiology, Department of Cardiac, Thoracic, Vascular Sciences and Public Health, Padua University Hospital and University of Padua, Via Giustiniani 2, Padua 35128, Italy; Unit of Biostatistics, Epidemiology and Public Health, Department of Cardiac, Thoracic, Vascular Sciences and Public Health, Padua University Hospital and University of Padua, Via Giustiniani 2, Padua 35128, Italy; Cardiology, Department of Cardiac, Thoracic, Vascular Sciences and Public Health, Padua University Hospital and University of Padua, Via Giustiniani 2, Padua 35128, Italy

**Keywords:** Myocarditis, Prognosis, Death, Heart transplant, Machine learning, Random forest

## Abstract

**Aims:**

Identifying early risk predictors in myocarditis is clinically relevant, as patients’ outcomes may be very diverse. We aimed to explore predictors of death and heart transplant (HTx) in a large single-centre cohort of adult patients with myocarditis using a machine learning (ML) technique.

**Methods and results:**

We retrospectively enrolled consecutive adult patients with biopsy-proven or clinically suspected myocarditis, collecting clinical, laboratory, and imaging data, both at diagnosis and during follow-up. A predictive model of death/HTx was developed using random forest (RF), ranking covariates according to their predictive accuracy. We included 938 patients (median age 36 years, 69% male) with clinically suspected (*n* = 549) or biopsy-proven (*n* = 389) myocarditis. During follow-up, 35 patients died, and 26 underwent HTx. The most important variables in predicting survival were NYHA class (variable importance, VIMP, 10%) LVEF (3.6%) and clinical presentation (2.5%) at diagnosis, histological type of myocarditis on endomyocardial biopsy (EMB)(2.9%), anti-endothelial cell antibodies (0.6%), and anti-nuclear antibodies (0.4%) positivity. Overall, the predictive accuracy of our RF model was good (89.2%, 95% C.I. 86.1–92.3).

**Conclusion:**

Based on a ML approach, we found, with good predictive accuracy, that advanced NYHA class, reduced LVEF and heart failure at diagnosis, and giant cell myocarditis on EMB are predictors of worse prognosis in adult patients with myocarditis.

## Graphical Abstract

**Graphical Abstract:**
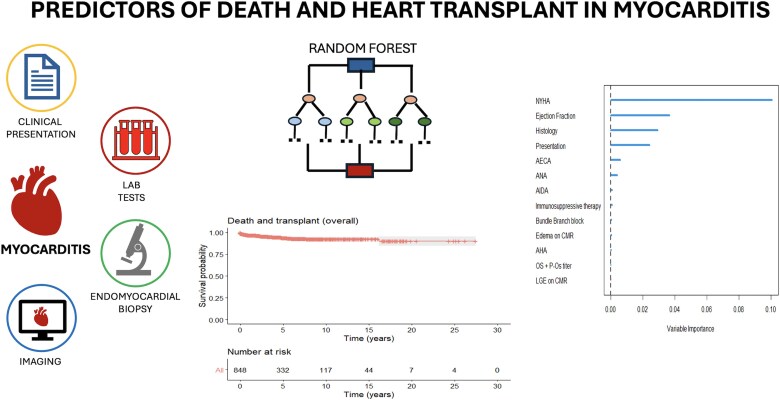

## Introduction

Myocarditis is an inflammatory disease of the myocardium characterized by heterogeneous disease course, ranging from *restitutio ad integrum* to progression to dilated cardiomyopathy and death/heart transplant (HTx).^1^ Identifying outcome predictors is of utmost clinical relevance as it could be a gatekeeper in patients’ management, possibly from a very early disease stage. The increasing number of potential clinical predictors has paved the way for the expanding application of artificial intelligence (AI) algorithms, especially for risk stratification,^2^ as it appears to outperform traditional regression models by producing a more flexible relationship among predictors and outcomes. We aimed to assess predictors of death and HTx in a large single-centre cohort of adult patients with biopsy-proven or clinically suspected myocarditis by using AI algorithms.

## Materials and methods

We included patients with biopsy-proven or clinically suspected myocarditis (strictly defined according to 2013 ESC and 1995 WHO criteria^1^) and collected clinical, laboratory, and imaging data, both at diagnosis and during follow-up. The study was approved by our Ethics Committee (protocol number 0021857).

Random survival forests (RF) were used to model the survival probabilities of patients. The model was trained using the following hyperparameters: the minimum number of observations required for each terminal node was set to 15, striking a balance between model complexity and stability; the mtry was set to 4, and we chose 10,000 iterations to ensure stable and accurate predictions. To assess the significance of the predictor variables in the RF model, the permutation method of variable importance (VIMP) was used. To improve clinical interpretability, by showing the marginal effect of the variable on predicted survival, partial dependence plots were generated considering the probability of predicted survival at 3 years, as it corresponded approximately to the median follow-up time of the study. The model was estimated using the randomforestSRC package within the R software.^3^

## Results

**Figure 1:**
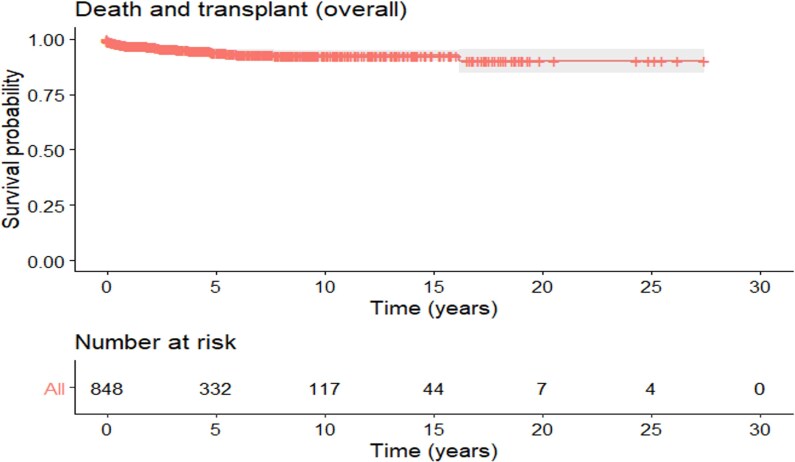
Overall survival of the studied cohort by Kaplan–Meier analysis.

We included in the analysis 938 patients (median age 36 years, IQR 25–48 years, 69% male) with clinically suspected (*n* = 549) or biopsy-proven myocarditis (*n* = 389, lymphocytic myocarditis in 58% of cases). The characteristics of the studied cohort are detailed in ***Table 1***. During 3 year follow-up (IQR 1–7 years), 35 patients (4%) died and 26 (3%) underwent HTx. Overall survival was 97% at 1 year and 93.9% at 5 years (***Figure 1***).

**Table 1:** Patients’ characteristics at the time of myocarditis diagnosis. Data are median (IQR) for continuous variables and absolute numbers (percentages) for categorical variables

	Missing data	*n* = 938
Age, years	—	36 (25–48)
Gender, male	—	646 (69)
NYHA class at diagnosis	8	
I, II		836 (90)
III–IV		94 (10)
Signs of left-sided HF	7	183 (20)
Signs of right-sided HF	8	65 (7)
Cardiac rhythm	33	
Sinus rhythm		857 (95)
Atrial fibrillation		22 (2)
Other		26 83)
Bundle branch block	66	
Left		49 (6)
Right		53 (6)
Atrioventricular block	68	40 (5)
LVEDV echo, mL/m2	263	67 (56–83)
LVEF echo, %	111	54 (40–60)
AHA positivity	222	373 (52)
AIDA positivity	247	223 (32)
AECA positivity	296	19 (3)
ANA positivity	270	65 (10)
Oedema on CMR	327	334 (55)
LGE on CMR	334	509 (84)

AECA, anti-endothelial cell antibodies; AHA, anti-heart antibodies; AIDA, anti-intercalated disk antibodies; ANA, anti-nuclear antibodies; CMR, cardiovascular magnetic resonance; HF, heart failure; LGE, late gadolinium enhancement; LVEDV, left ventricular end-diastolic volume; LVEF, left ventricular ejection fraction.

**Figure 2:**
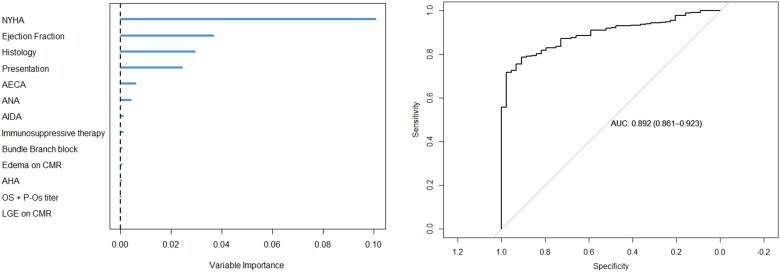
Left panel, variable importance plot according to the random forest algorithm. Right panel, ROC-curve showing the predictive accuracy of our machine learning model. AECA, anti-endothelial cell antibodies; AHA, anti-heart antibodies; AIDA, anti-intercalated disk antibodies; ANA, anti-nuclear antibodies; CMR, cardiovascular magnetic resonance; LGE, late gadolinium enhancement; NYHA, New York Heart Association; OS, organ specific; P-OS, partially organ specific.

The model evaluating the variables’ impact on predicting survival ranked them, based on VIMP, as follows (***Figure 2***): NYHA at diagnosis (10%, which means 10% reduction in the model's accuracy when NYHA class is permuted), LVEF (3.6%), histological type of myocarditis on EMB (2.9%), clinical presentation (2.5%), anti-endothelial cell antibodies (AECA, 0.6%), and anti-nuclear antibodies (ANA, 0.4%) positivity; anti-heart antibodies (AHA) showed VIMP close to zero. Oedema and late gadolinium enhancement (LGE) on CMR at diagnosis, as well as immune-suppressive therapy at follow-up, did not contribute to the model's accuracy. Partial dependence plots showed that three-year survival was lower for patients with advanced NYHA class (85% 3 year survival for NYHA III-IV vs. 97% for patients with NYHA class I-II), LVEF <40% (93% survival vs. 96% for LVEF >40%), heart failure presentation (94% vs. 96% for arrhythmic and infarct-like presentation), giant cell myocarditis (GCM) (83% vs. 95% for other histological myocarditis types and 96% for clinically suspected myocarditis), and auto-antibodies positivity (89% for AECA positivity vs. 96% for AECA negativity; 94% for ANA positivity vs. 96% for ANA negativity). Overall, the accuracy of our RF model was good, with an OOB AUC of 89.2% (95%, C.I. 86.1–92.3) (***Figure 2***).

## Discussion

The most significant predictors of death and HTx in our cohort of myocarditis patients were advanced NYHA class and lower LVEF at diagnosis and the histological type of myocarditis on endomyocardial biopsy (EMB). Advanced NYHA class at diagnosis strongly correlated with lower 3 year survival, in keeping with previous studies^4–5^ showing a threefold increase in risk of death/HTx for NYHA class III-IV patients.^6^ Likewise, LVEF at diagnosis, though slightly less impactful than NYHA class, remained a robust long-term outcome predictor, as previously shown by our group and others.^4,5^ Finally, the histological type of myocarditis correlated with outcome,^7^ particularly GCM, previously associated with up to 48% 5 year mortality.

Interestingly, as opposed to previous findings,^5^ autoimmune markers (AHA/ANA) were not strongly associated with outcome. This may be explained by the inclusion, in this study, of biopsy-proven virus-negative AHA-positive myocarditis patients receiving immune-suppression, as indicated by international recommendation; the previously described adverse predictive role of auto-antibodies positivity may therefore be lost by the effect of immune-suppressive treatment, known for its beneficial effects on clinical and functional status.^8^

As opposed to earlier studies, CMR imaging markers like LGE and oedema were not predictive of adverse outcome^9^; this could be explained by the under-use of CMR in patients with fulminant or heart failure presentations, known to have worse outcome, as also shown in our study.

We believe that the machine learning (ML) approach has provided advantages over traditional statistical models by better handling complex relationships and multiple variables,^2^ also in view of the relatively small number of adverse events at follow-up (6.5%) in our cohort. By using RF, which is better suited to contexts with a limited number of events and multiple predictors (i.e. factors potentially compromising reliability of a Cox model^10^), we could focus on identifying relevant predictors while reducing the risk of overfitting. This study's model has highlighted predictors previously missed by standard multi-variable analysis, in a similar cohort of patients,^5^ like heart failure presentation. Moreover, myocarditis is intrinsically a heterogeneous disease, with variable course and different treatment strategies that have been implemented over the past two decades; this may introduce a bias when interpreting the study's results. However, the RF approach by overcoming group comparisons and providing a tool that is less affected by outliers and more flexible on statistical assumptions on data distribution may have been advantageous in our cohort, where both clinically suspected and EMB-proven myocarditis, known to have different disease course, were included. Immune-suppressive treatment, whose indication strictly followed ESC recommendations,^7^ was started in eligible patients following a safety check-list, ruling out underlying on-going infections and malignancies; time required to start immune-suppression could therefore vary from patient to patient, although it was generally started as inpatient. Taking into account the time-dependent nature of this variable would have required a different analysis model, based on time-dependent covariates or landmark analysis. However, the number of events among patients treated with immunosuppressive therapy was limited and therefore insufficient to allow a robust time-dependent modelling strategy.

As opposed to recent studies using multimodal data and different AI algorithms to predict major adverse cardiovascular events in myocarditis,^11,12^ ours was not meant to be a comparative study between different ML classifiers. The aim of the study was to explore predictors of adverse outcomes in a cohort of patients with myocarditis using a ML approach commonly used in clinical research. Random forests were chosen because they offer a robust, well-established framework for identifying relevant predictors in clinical datasets, particularly in contexts with a relatively low number of events in relation to the number of candidate predictors. Their relative stability and direct assessment of VIMP make them particularly well suited to clinical analyses, where interpretability and robustness take priority over marginal gains in predictive performance.^13–15^ The present analysis was based on a single ML method, consistent with the aim of identifying clinically meaningful predictors rather than benchmarking different algorithms. The findings of the present study should also be interpreted in light of its exploratory nature and of its single-centre design. External validation in independent cohorts will be needed before the identified predictors can be translated into a clinical decision-support tool.

## Conclusions

We have developed an overall accurate ML approach showing that advanced NYHA class, reduced LVEF and heart failure at diagnosis, and GCM on EMB are predictors of worse outcome in a large single-centre cohort of clinically suspected or biopsy-proven myocarditis. Further studies are anyway needed to test the accuracy of our exploratory model on external cohorts, to possibly implement its use in clinical practice.

## Data Availability

Data are available upon reasonable request to the corresponding author.

## References

[1] Caforio ALP, Pankuweit S, Arbustini E, Basso C, Gimeno-Blanes J, Felix SB, Fu M, Helio T, Heymans S, Jahns R, Klingel K, Linhart A, Maisch B, McKenna W, Mogensen J, Pinto YM, Ristic A, Schultheiss H-P, Seggewiss H, Tavazzi L, Thiene G, Yilmaz A, Charron P, Elliott PM. Current state of knowledge on aetiology, diagnosis, management, and therapy of myocarditis: a position statement of the European Society of Cardiology working group on myocardial and pericardial diseases. Eur Heart J 2013;34:2636–2648. 23824828 10.1093/eurheartj/eht210

[2] Goldstein BA, Navar AM, Carter RE. Moving beyond regression techniques in cardiovascular risk prediction: applying machine learning to address analytic challenges. Eur Heart J 2017;38:1805–1814. 27436868 10.1093/eurheartj/ehw302 PMC5837244

[3] Ishwaran H, Kogalur UB. Fast Unified Random Forests for Survival, Regression, and Classification (RF-SRC) [R package manual version 3.2.0/3.2.2]. 2023. Available from: https://cran.r-project.org/package=randomForestSRC.

[4] Anzini M, Merlo M, Sabbadini G, Barbati G, Finocchiaro G, Pinamonti B, Salvi A, Perkan A, Di Lenarda A, Bussani R, Bartunek J, Sinagra G. Long-term evolution and prognostic stratification of biopsy-proven active myocarditis. Circulation 2013;128:2384–2394. 24084750 10.1161/CIRCULATIONAHA.113.003092

[5] Baritussio A, Schiavo A, Basso C, Giordani AS, Cheng C-, Pontara E, Cattini MG, Bison E, Gallo N, De Gaspari M, Carturan E, Thiene G, Tarantini G, Plebani M, Rizzo S, Gregori D, Iliceto S, Marcolongo R, Caforio ALP. Predictors of relapse, death or heart transplantation in myocarditis before the introduction of immunosuppression: negative prognostic impact of female gender, fulminant onset, lower ejection fraction and serum autoantibodies. Eur J Heart Fail 2022;24:1033–1044. 35377503 10.1002/ejhf.2496

[6] Kindermann I, Kindermann M, Kandolf R, Klingel K, Bültmann B, Müller T, Lindinger A, Böhm M. Predictors of outcome in patients with suspected myocarditis. Circulation 2008;118:639–648. Erratum in: Circulation 2008 Sep 16;118(12): e493. 18645053 10.1161/CIRCULATIONAHA.108.769489

[7] Schulz-Menger J, Collini V, Gröschel J, Adler Y, Brucato A, Christian V, Ferreira VM, Gandjbakhch E, Heidecker B, Kerneis M, Klein AL, Klingel K, Lazaros G, Lorusso R, Nesukay EG, Rahimi K, Ristić AD, Rucinski M, Sade LE, Schaubroeck H, Semb AG, Sinagra G, Thune JJ, Imazio M, Arbelo E, Basso C, Adamo M, Aktaa S, Ammirati E, Anderson L, Arbustini E, Bobbio E, Boriani G, Brida M, Byrne RA, Caforio ALP, Dan G-A, Domínguez F, Fredericks S, Gulati G, Ibanez B, James S, Kharlamov A, Klaassen S, Kluin J, Koskinas KC, Kuchynka P, Kunadian V, Landmesser U, Lip GYH, Maisch B, Marelli-Berg F, Martin P, McEvoy JW, Mihaylova B, Mindham R, Moelgaard I, Mohiddin SA, Nielsen JC, Pasquet AA, Peretto G, Pilichou K, Piriou N, Prescott E, Rakisheva A, Rocca B, Rossello X, Sannino A, Seidel F, Tanner FC, Tomkowski WZ, Vaartjes I, Van Linthout S, Vrints C, Wojnicz R, Zeppenfeld K, Banushi AD, Chettibi M, Sisakian HS, Musayev O, Paelinck BP, Begić A, Daskalov Y, Skoric B, Ioannides M, Palecek T, Rossing K, Ghareeb HS, Hinto U, Kupari M, Berthelot E, Agladze V, Gerull B, Kasiakogias A, Vágó H, Ingimarsdóttir IJ, Joyce E, Goland S, Fabris E, Mukarov MA, Bytyçi I, Mirrakhimov E, Kamzola G, Abirached NJ, Smer AM, Mizariene V, Codreanu A, Felice T, Vataman EB, Soufiani A, Bijvoet GP, Mitevska I, Ravnestad H, Kamiński K, Cardim N, Geavlete O, Troiani E, Zdravkovic M, Dankova M, Čerček AČ, Domínguez F, Arefalk G, Haaf P, Marjeh MYB, Kilicaslan B, Cherniuk S, Mohiddin SA, Kevorkov A; ESC Scientific Document Group. 2025 ESC guidelines for the management of myocarditis and pericarditis. Eur Heart J 2025;46:3952–4041. 40878297 10.1093/eurheartj/ehaf192

[8] Frustaci A, Russo MA, Chimenti C. Randomized study on the efficacy of immunosuppressive therapy in patients with virus-negative inflammatory cardiomyopathy: the TIMIC study. Eur Heart J 2009;30:1995–2002. 19556262 10.1093/eurheartj/ehp249

[9] Georgiopoulos G, Figliozzi S, Sanguineti F, Aquaro GD, Di Bella G, Stamatelopoulos K, Chiribiri A, Garot J, Masci PG, Ismail TF. Prognostic impact of late gadolinium enhancement by cardiovascular magnetic resonance in myocarditis. A systematic review and meta-analysis. Circ Cardiovasc Imaging 2021;14:e011492. 33441003 10.1161/CIRCIMAGING.120.011492

[10] Peduzzi P, Concato J, Kemper E, Holford TR, Feinstein AR. A simulation study of the number of events per variable in logistic regression analysis. J Clin Epidemiol 1996;49:1373–1379. 8970487 10.1016/s0895-4356(96)00236-3

[11] Pfeffer A, Pezel T, Toupin S, Garot P, Hovasse T, Sanguineti F, Di Lena C, Renard C, Tribouilloy C, Hamzi K, Goncalves T, Dillinger JG, Henry P, Bohbot Y, Garot P. Machine learning using cardiovascular magnetic resonance to predict cardiovascular events in patients with acute myocarditis. Eur Heart J 2023;44:ehad655.1825.

[12] Baj G, Kazaj PM, Schutze J, Bernhard B, Siontis G, Kwong RY, Shiri I, Graeni C. Major adverse cardiovascular events prediction in patients with suspected myocarditis using multimodal data and artificial intelligence. Eur Heart J 2025;46:ehaf784.232.

[13] Breiman L . Random forests. Mach Learn 2001;45:5–32.

[14] Hastie T, Tibshirani R, Friedman J. Random forests. In: The Elements of Statistical Learning. *Springer Series in Statistics*. New York, NY: Springer; 2009. p587–604.

[15] Barreñada L, Dhiman P, Timmerman D, Boulesteix A-L, Van Calster B. Understanding overfitting in random forest for probability estimation: a visualization and simulation study. Diagn Progn Res 2024;8:14. 39334348 10.1186/s41512-024-00177-1 PMC11437774

